# Hydrogenated Planar Aluminum Clusters: A Density Functional Theory Study

**DOI:** 10.3390/molecules30061389

**Published:** 2025-03-20

**Authors:** Changhong Yao, Meijiao Wang, Lianzhen Cao

**Affiliations:** School of Physics and Electronic Information, Weifang University, Weifang 261061, China

**Keywords:** cluster, structure, density functional theory

## Abstract

The low-lying energy structures of small planar aluminum clusters Al_n_ (n = 3–6, 8–10), hydrogenated small planar aluminum clusters Al_n_H_m_ (n = 3–8, m = 1–2) and the lowest-energy structure of Al_n_H_m_ (n = 6–10, m = 0–2) are determined by density functional theory (DFT) calculations. Many stable planar structures have been found; some are consistent with the reported ones, and some are new configurations. The preservation of planar cluster structures has been observed during the dissociative adsorption of H_2_.Hydrogen is adsorbed at different positions on planar aluminum clusters. Dissociative adsorption configurations of the planar structure and lowest-energy structure experienced a decrease in hydrogen adsorption energy with an increase in cluster size. Among the clusters we calculated, Al_4_H_1_ and Al_4_H_2_ have the highest HOMO-LUMO gap, indicating that they may be more abundant than other clusters. The geometric structure and electronic properties of these clusters are also discussed.

## 1. Introduction

Hydrogen is a clean and renewable form of energy, which will not pollute the environment, so it is an ideal substitute for oil and is expected to become one of the main energy sources in the future. Finding an ideal hydrogen storage medium is a major problem that limits the wide application of hydrogen in industry and life. Solid-state hydrogen storage is a relatively safe method [1]. However, the hydrogen storage medium with good reversibility at ambient temperature is not satisfactory. It is necessary to find new hydrogen storage materials [2]. As a solid-state system, the interaction between atomic clusters and hydrogen is of great value for elucidating the mechanism of solid-state hydrogen storage. Therefore, as potential hydrogen storage materials, different types of clusters are worth exploring more through extensive research. Such research is expected to be applied in the fields of catalysis and hydrogen storage materials [3].

Aluminum cluster hydrogen storage, as a new type of hydrogen storage method, has unique advantages. Firstly, aluminum clusters can achieve high hydrogen storage density through specific hydrogen storage mechanisms such as charge polarization [4,5,6]. This means that aluminum clusters can store more hydrogen gas at the same volume or mass, thereby improving hydrogen storage efficiency. Secondly, some aluminum cluster hydrogen storage systems have reversed [7], which means they can achieve hydrogen adsorption and release under appropriate conditions. This is crucial for the practical application of hydrogen energy, as reversibility means that hydrogen storage materials can be reused, reducing costs and improving energy efficiency. Again, aluminum is a relatively inexpensive, lightweight material, so the cost of aluminum cluster hydrogen storage materials is relatively low. This helps to reduce the overall cost of hydrogen energy and promote its commercialization process. In terms of application prospects, aluminum cluster hydrogen storage is expected to be applied in fields, such as fuel cells, hydrogen vehicles, and hydrogen power plants. With the continuous development of the hydrogen energy industry, aluminum cluster hydrogen storage technology will become one of the key technologies to promote the commercial application of hydrogen energy.

Recent interest in experimental and theoretical studies has been focused on the adsorption of hydrogen atoms on aluminum-based clusters [8,9,10,11,12]. Experimentally, Vanbuel et al. investigated the interaction of hydrogen with Al_n_Rh_2_^+^ clusters (n = 10–13) using mass spectrometry and multi-photon dissociative infrared spectroscopy (IRMPD). By comparing the results with DFT calculations, they found that a single hydrogen molecule dissociated at n = 10,11 and absorbed at n = 12, 13 [12]. Vanbuel et al. also discovered through the same experimental techniques that vanadium doping enhances the reactivity of aluminum clusters to hydrogen [13].Through a flow reactor experiment with first-principles theoretical investigations, Al_4_H_7_^−^ is shown to have the ability to bond with ionic partners to form stable hydrides through the addition of an alkali atom [XAl_4_H_7_ (X = Li-Cs)] [14]. Through the study of photoelectron spectroscopy and theoretical calculations of Al_3_H_n_^−^ (n = 1–9) clusters, Xu et al. discovered three modes of hydrogen binding to Al_3_: terminal, bridging, and capping [15]. In theoretical research, the study of hydrogen adsorption on aluminum-based clusters has attracted widespread attention. The hydrogen molecule uses its occupied orbit to react with the appropriate electron-deficient sites of the Al_n_ (n = 5–7) clusters to form hydrogenated aluminum clusters, studied by Maatallah et al. using DFT [16].The bond order based on energy density for hydrogenated aluminum clusters (Al_n_H_m_, n = 1–8 and m = 1–2) was calculated by using an electronic stress tensor by Ichikawa et al. [4]. Ma et al. reported Al_4_Si_2_H_16_ has the highest hydrogen capacity (8.9 weight%) and moderate bonding strength (−0.55 eV per H_2_) by using the evolutionary algorithm combined with ab initio computations [2]. The interaction between the H-1s orbital and only certain molecular orbitals of Al_13_ is responsible for the binding of hydrogen in the Al_13_H, which is analyzed in the framework of DFT under the local density approximation (LDA) level [17]. Maatallah et al. reported that Al_n_H_n+2_ (n = 4–6) no longer has a closed structure due to the presence of bridged hydrogen atoms, and their calculations are based on the theoretical level of B3LYP/6-311+G(d,p), BPW91/6-311G(d,p) and B3LYP/6311+G(3df,2p) [18]. After using DFT calculations, Duque et al. proposed that Al_13_H has a closed electron shell, which makes the cluster very stable [19]. Kawamura et al. found that due to the large binding energy and the highest occupied–lowest unoccupied molecular orbital (HOMO-LUMO) gap, Al_7_H and Al_13_H can be considered as magic clusters [20]. Through ab initio calculations of Al_3_BH_2n_ (n = 0–6), Muz et al. found that the more hydrogen molecules absorbed, the greater the stability of boron-doped Al_3_ hydrogenated clusters [21]. Under the DFT B3LYP level, Charkin et al. studied hydrogenated aluminum clusters Al_13_H_p_, Al_44_H_n_ and Al_89_H_m_ (p = 1–12, n = 2–44 and m = 15–63). They found that the geometric distortions of the Al_44_H_n_ and Al_89_H_m_ clusters grow with rising n and m [22]. Using DFT and a modified G3(MP2)-RAD procedure, the interaction between hydrogen molecules and aluminum clusters Al_12_X (X = Mg, Al, Si) was studied by Henry et al. They found the barriers for H_2_ desorption from the di hydrogenated clusters are generally quite substantial [23]. Gandhi et al. reported the theoretical calculations of the hydrogen desorption energies of Al_n_H_3n_ clusters based on DFT. They found Al_n_H_3n_ clusters of sizes n = 8–16 have desorption energies in the range 0.6–0.4 eV per H_2_, which is suitable for hydrogen storage applications [24]. Kiran et al. proposed an electron counting rule to predict magic clusters consisting of hydrogen and aluminum atoms [25]. Pino et al. reported transition states and reaction paths for a hydrogen molecule dissociating on small aluminum clusters using DFT [26]. Using the established correlated ab initio methods, MP2 and CCSD(T), in conjunction with the augmented correlation consistent basis sets up to aug-ccpVTZ, Moc et al. studied the interaction of the Al_1_^−^ anion cluster with H_2_ [27]. Yamamura et al. studied Al_12_H_12_^2−^ using ab initio molecular orbital calculations. They found covalent bonding Al_12_H_12_^2−^ clusters cannot be reproduced by the Woods–Saxon model [28]. Ichikawa et al. reported on the structures of aluminum hydrides derived from a tetrahedral aluminum (Al_4_) cluster using ab initio quantum chemical calculation. They found that the stability of hydrides is more stable as the number of hydrogen atoms increases, but the stability of the AlH bond decreases [29]. DFT calculations were carried out to study the linear polymeric Al_n_H_3n_ (n = 1–12) clusters and cage Al_n_H_3n_ (n = 6, 8, 10, and 12) clusters by Xu et al. [5]. Varano et al. investigated how Al_13_ cluster dimmers can be formed with or without a bridging hydrogen [30].

In the above studies, the shapes of aluminum clusters are mostly three-dimensional structures, with few two-dimensional planar structures. The shape of aluminum clusters has an important impact on the adsorption of hydrogen atoms. However, there is little research on the mechanism of adsorption of hydrogen atoms by planar aluminum clusters. With an increase in cluster size, the geometric structure of clusters gradually tends to be bulk materials. Therefore, the structure of most clusters is three-dimensional, while two-dimensional planar structures mostly exist in some small clusters. There are also some reports on planar clusters in the literature, for instance, planar B_n_P_2_ (n = 1–7) [31], the planar Ga_5_N_5_ cluster [32] and the quasi-planar B_56_ boron cluster [33]. For small aluminum clusters, some planar surfaces have also been reported [34,35,36,37,38].

Planar aluminum clusters typically have a larger specific surface area than three-dimensional clusters due to their two-dimensional structure. A larger specific surface area means more adsorption sites, which is beneficial for the adsorption and storage of hydrogen. Due to the increase in specific surface area, planar aluminum clusters can adsorb more hydrogen molecules, thereby achieving higher hydrogen adsorption density. This is crucial for improving hydrogen storage efficiency and energy density. The two-dimensional structure of planar aluminum clusters may make the adsorption process of hydrogen molecules more controllable. By adjusting the structure and composition of planar clusters, the adsorption position and energy of hydrogen molecules can be optimized, thereby achieving more efficient hydrogen storage and release. Hydrogen desorption is one of the important performance indicators of hydrogen storage materials. Planar aluminum clusters, due to their unique structure, may have better hydrogen desorption performance, meaning they can more easily release hydrogen under appropriate conditions, meeting the needs of practical applications. In this study, we investigated the planar structure of stable small aluminum clusters in detail and studied the adsorption of hydrogen atoms on planar aluminum clusters. We compare the adsorption of hydrogen atoms on planar clusters and the lowest-energy clusters. These studies are beneficial supplements to the adsorption of hydrogen atoms on aluminum clusters and provide theoretical support for possible planar hydrogen storage materials.

The rest of this paper is organized as follows. In Section 2, we present and discuss the obtained results of small planar Al_n_ clusters and the adsorption of hydrogen atoms on the stable planar Al_n_ clusters. In Section 3, we sketch the main computational method used in the calculation. Finally in Section 4, we briefly summarize our calculations.

## 2. Result and Discussion

### 2.1. Structures of the Small Planar Al_n_ (n = 3–10) Clusters

**Figure 1:**
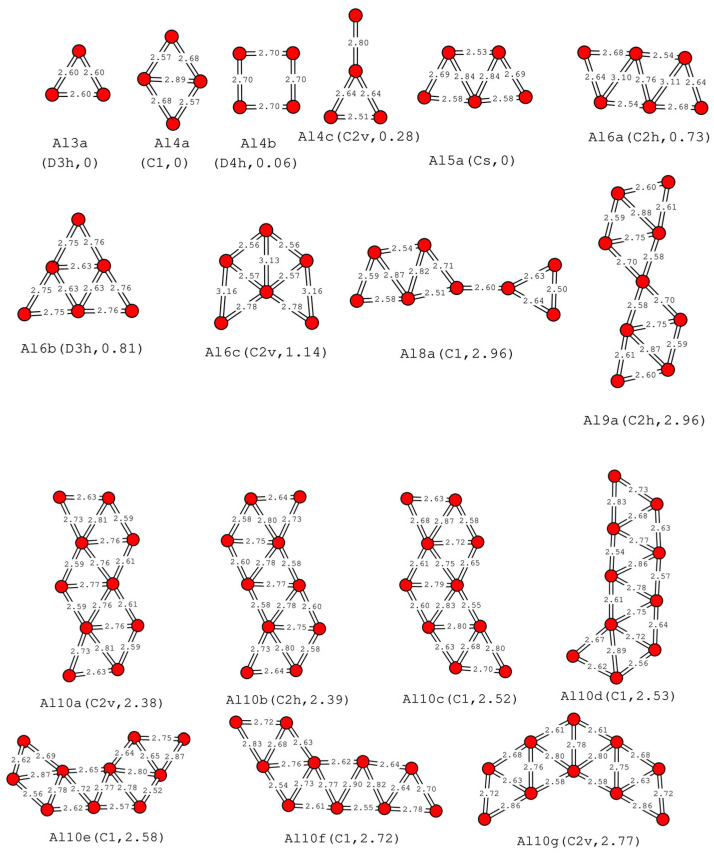
The low-lying energy planar structures and their isomers of Al_n_ clusters (n = 3–6, 8–10). The name suffix of the lowest energy planar structure is a, and the name suffix of the isomer is b or c. The point group symmetries, the relative energy of the isomer relative to the lowest-energy structure are given in parentheses. The unit of energy is eV, and the unit of length is angstroms.

The planar Al_n_ clusters (n = 3–10) are investigated via DFT. Many isomeric structures are obtained. In this section, only planar structures are introduced, as shown in Figure 1. The three-dimensional structures will be introduced later. The vibrational frequencies have been calculated for the clusters in Figure 1. For clarity, Al_3_ represents clusters, Al3a is the name of the structure in Figure 1, and the following are similar.

In our calculations, the stable planar structure of Al_3_ is an equilateral triangle, as shown in Figure 1, with a side length of 2.60 Å and D_3h_ symmetry (the threshold value of point group symmetry is 0.1 Å, the same below), which are consistent with previous reports [35,36,38,39,40,41,42]. Al_4_ has several stable planar structures. These structures are named Al4a, Al4b, and Al4c. Al4a has the lowest energy among planar Al_4_ clusters, followed by Al4b and Al4c. The following naming methods are the same. The planar structure with the lowest energy in our calculation is a quadrilateral with C_1_ symmetry (Al4a in Figure 1). Similar structures have been reported by Jones [35], but in Jones’ research, this similar structure is not the structure with the lowest energy but, rather, has higher energy than a rhombus structure. Compared to the widely reported rhombus structure [34,35,36,37,38,39,40,41,42,43,44], our calculation shows that Al4b in Figure 1 is a rectangular planar structure with high D_4h_ symmetry, with an energy of 0.06 eV higher than Al4a. Al4c in Figure 1 is a C_2v_ symmetric structure, and its shape is a capped atom on the vertex of the isosceles triangle. The planar structure of Al_5_ we obtained has been reported in the literature [16,34,35,36,37,38,39,40,41,42,44]. The planar structure is C_s_ symmetric in our calculations. Al6a is a C_2h_ structure that has been reported [16,34,35,36,37]. Al6b is a D_3h_ structure with higher symmetry, with an energy of 0.08 eV greater than Al6a, which has also been reported by Maatellah et al. [16]. Al6c is a C_2v_ symmetric new structure, with an energy increase of 0.41 eV compared to Al6a. We have not yet searched for the two D_2h_ symmetric Al_6_ planar structures reported by Pettersson et al. [34]. The Al_7_ plane structure with a hexagonal shape embedded with an atom at the center has been reported [35,37]. However, we have not yet found any stable planar structure of Al_7_ in our extensive search. We have discovered an Al_8_ planar structure formed by bridging Al3a and Al5a. We also found an Al_9_ planar structure formed by two Al5a sharing one atom, with C_2h_ symmetry. Unlike the rarity of Al_7_, Al_8_, and Al_9_ planar structures, there are a large number of Al_10_ planar structures (from Al10a to Al10g) present. The common feature of these structures is that their nearest neighboring atoms form the Al_3_ structure. As the number of atoms increases, the planar structure will become more and more unstable. For the reliability of Al_10_ with so many planar structures, we used ORCA software(version 4.2.1) [45,46,47] to calculate the structure again. **Revised Perdew-Burke-Ernzerhof** (RPBE) functional and def2-TZVP [48] basis function are used in ORCA. We found that the structures of Al10f and All10g have large deformation, Al10d has slight deformation, and other Al10a, Al10b, Al10c, and Al10e remain unchanged in the original plane configuration. This shows that these planar structures are relatively stable. We have not yet discovered the Al_10_ structure reported by Ueno et al. [37]. Their structure also has the characteristic of nearest neighbor atoms forming triangles.

### 2.2. Structures of One or Two Hydrogen Atoms Adsorbed on Small Planar Al_n_ Clusters

**Figure 2:**
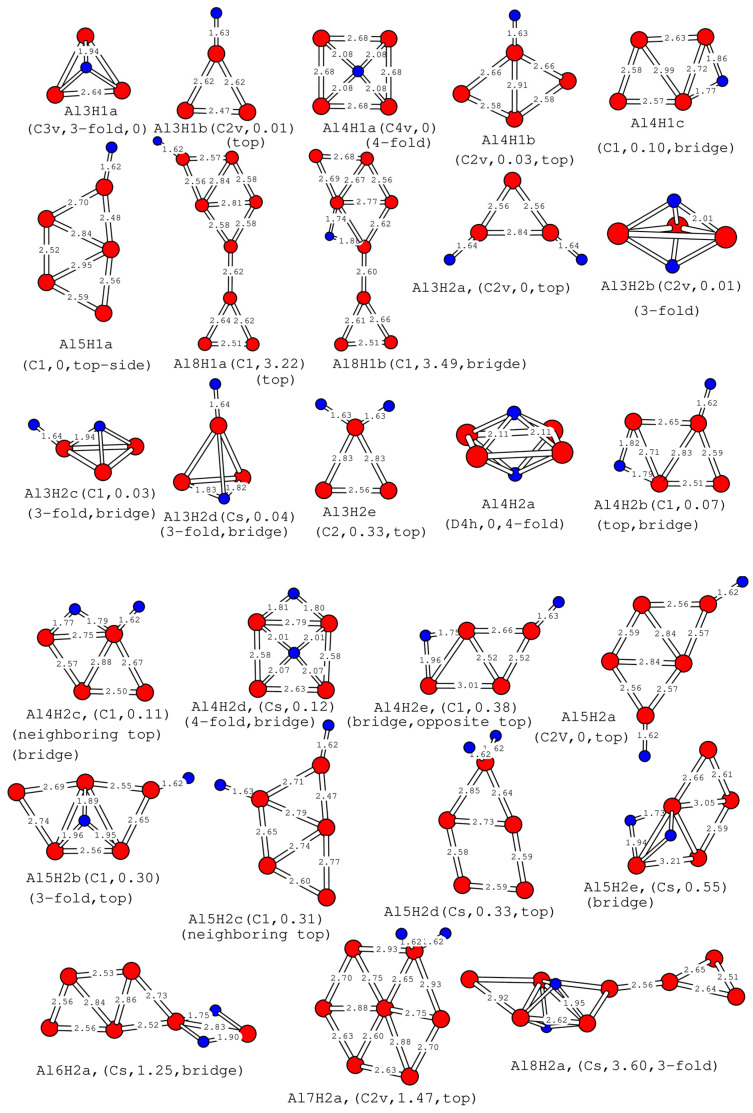
The low-lying energy structures of Al_n_H_m_ clusters (n = 3–8, m = 1–2) and their isomers. Location of H is represented by 3-fold,4-fold, neighboring, opposite, bridge and top in parentheses. The red big ball represents aluminum atoms, and the blue small ball represents hydrogen atoms.

Add one or two hydrogen atoms to the planar aluminum cluster obtained above. The position of the hydrogen atom is random, and there are at least fifty such initial configurations. After DFT optimization, abandoning the case where Al_n_ is no longer a plane in stable results, which means keeping Al_n_ basically undeformed, allows the hydrogen atom to exist out of the Al_n_ plane. The results of the atomic structure of one and two hydrogen atoms on small planar Al_n_ clusters are shown in Figure 2. The vibrational frequencies have been calculated for the clusters in Figure 2.

In Figure 2, hydrogen in the Al3H1a structure (C_3v_ symmetry) is located in a high coordination number (three-fold) site, which is consistent with the report of Kawamura et al. [49]. In the report [49], there is also a sub-energy structure where the hydrogen atom is located at the bridge site of Al_3_. However, in our calculations, we have not yet found a stable structure where the hydrogen atom is located at the bridge site. Al3H1b is another stable structure, with a hydrogen atom located at the top of an aluminum atom. This structure has an energy of 0.01 eV higher than Al3H1a and exhibits C_2v_ symmetry. Hydrogen in the Al4H1a structure (C_4v_ symmetry) is located at a high coordination number (four-fold), where the Al_4_ in Al4H1a is a square. Hydrogen in the Al4H1b structure (C_2v_ symmetry, 0.03 eV higher than Al4H1a in energy) is located at the top of an aluminum atom, in which the Al_4_ in Al4H1b is an approximate diamond shape. In Al4H1c, the hydrogen atom is located at the bridge site of the aluminum quadrilateral, which has also been reported by Kawamura et al. [49]. In Al5H1a, the hydrogen atom is located at the top of an aluminum atom in the Al5a structure in Figure 1, which has also been reported by Kawamura et al. [49]. Adding a hydrogen atom to the top of an aluminum atom on the Al8a structure in Figure 1 yields Al8H1a. Adding it to the bridge between the two aluminum atoms yields Al8H1b, which is 0.27 eV higher in energy than Al8H1a.

When two hydrogen atoms are added to Al_3_, the following situations occur: (1) Two hydrogen atoms are located at the tops of the two hydrogen atoms, forming a C_2v_ symmetric Al3H2a. (2) The relative positions of the two hydrogen atoms have three coordination numbers (two three-fold) with A_3_, respectively, forming Al3H2b with energy 0.01 eV higher than Al3H2a. (3) One hydrogen atom is three-fold, and the other is located at the top position, resulting in Al3H2c. (4) One hydrogen atom at the top position and the other at the bridge position yield Al3H2d. (5) Both hydrogen atoms are at the top of the same aluminum atom, resulting in Al3H2e. The structure of Al_4_H_2_ is similar to that of Al_3_H_2_. They are as follows: Al4H2a with two hydrogen atoms, both four-fold, Al4H2b with one bridge and one top, Al4H2c and Al4H2e with one bridge and one top, and Al4H2d with one four-fold and one bridge. Similarly, for Al_5_H_2_, the different combinations of two hydrogen atoms at the top position, four-fold position, and bridge position form several different configurations from Al5H2a to Al5H2e. In the above configurations, Al3H2b, Al3H2d, Al4H2b, Al4H2c, and Al5H2a have all been reported in the literature [49]. Maatellah et al. also reported on Al5H2a [16]. Both hydrogen atoms in Al6H2a are located at bridge sites. In Al7H2a, both hydrogen atoms are located at the top site of the same atom. It should be emphasized that the aluminum atom Al_7_ in Al7H2a has a hexagonal structure with an aluminum atom embedded in the center. This structure has been reported [35,37], but this configuration of Al_7_ cannot exist stably in our calculations. Both hydrogen atoms in Al8H2a are located at the three-fold sites. Our calculations suggest that two hydrogen atoms are likely to be dissociated on these clusters, which is an agreement with the result of Kawamura et al. [49].

When one or two hydrogen atoms are adsorbed on planar aluminum clusters, the geometric structure of planar aluminum clusters changes minimally. In the calculation, we did not force the fixed plane structure. The reason for this result is that we randomly selected more than 20 possibilities for the initial positions of hydrogen atoms on the plane structure and then calculated at least 20 different initial hydrogen positions. Among these results, those with almost the same structure of planar aluminum clusters are selected. Both Al_9_ and Al_10_ planar structures were deformed after hydrogen adsorption, so they were abandoned. In the process of hydrogen adsorption, the cluster structure remains unchanged, which is consistent with the conclusion of hydrogen adsorption on Ag_n_Cr (n = 1–12) [1].

### 2.3. Lowest-Energy Structures of Al_n_H_m_ (n = 6–10, m = 0–2)

**Figure 3:**
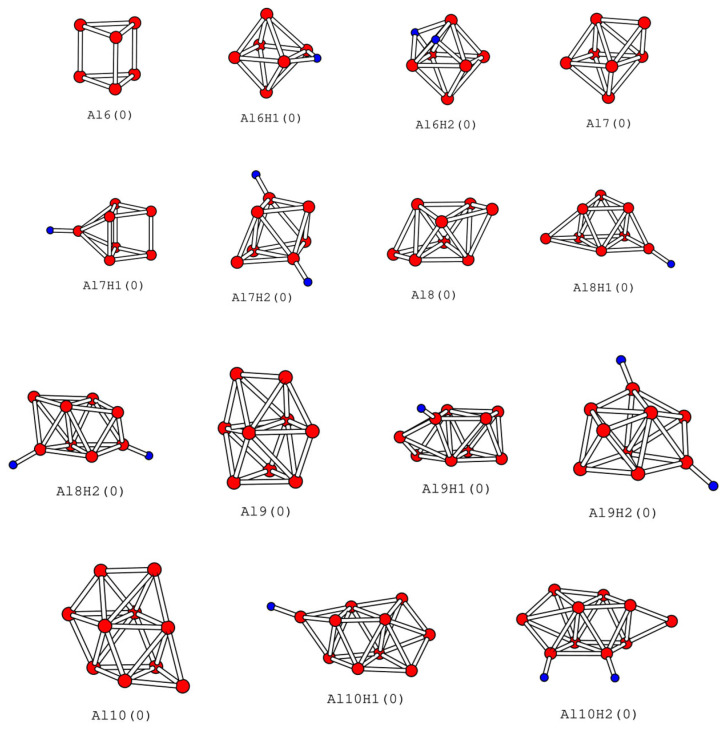
The lowest-energy structures of Al_n_H_m_ clusters (n = 6–10, m = 0–2). The relative energy of the isomer relative to the lowest-energy structure is given in parentheses.

When the number of aluminum atoms is greater than five, the global minimum energy structure of aluminum clusters is not planar. The real system with the lowest-energy structure is the most likely system to be prepared in experiments, so we also calculated the cluster system when n is greater than 5. By comparing the lowest-energy system with a planar structure, we can obtain information on the transition from a two-dimensional structure to a three-dimensional structure. The lowest-energy structures of pure aluminum clusters and aluminum clusters adsorbing one or two hydrogen atoms are listed in Figure 3. In our calculation, the lowest structure of Al_6_ is a triangular prism structure rather than a distorted octahedral [16,36,38,39,40,41,42,49] structure in this study. On the contrary, when one or two hydrogen atoms are absorbed, the lowest-energy structures of Al_6_H_1_ and Al_6_H_2_ are hydrogen atoms adsorbed on the octahedral aluminum cluster structure. The hydrogen atom in Al_6_H_1_ is at the bridge site rather than the three-fold site [16,49], while both hydrogen atoms in Al_6_H_2_ are adsorbed on the surface; the results are consistent with those of references [20,49]. Interestingly, the lowest structure of Al_7_ is to add an aluminum atom on one side of Al_6_ of the octahedron [38,39,40,49], while the lowest structure of Al_7_H_1_ is to add an aluminum atom on the quadrilateral side of Al_6_ of prism, and then the hydrogen atom is adsorbed on the increased aluminum atom [49]. The hydrogen atom in Al_7_H_2_ is located at the opposite top position. Al_8_ [42] and Al_8_H_2_ are close to a two-layer structure, while Al_8_H_1_ has large deformation relative to them. The structures of Al_9_, Al_9_H_1_, Al_9_H_2_ and Al_10_, Al_10_H_1_, Al_10_H_2_ show more changes, which shows that with the increase in the number of clusters, their structures are more and more diversified. Many structures of Al_9_ and Al_10_ [39,40,41,42] have been reported in the literature, but due to their complex configurations, it is not possible to compare them with our calculated results solely based on the images. Except for Al_6_H_1_ and Al_6_H_2_, the position of the hydrogen atom in other structures is at the top site, which shows that for the lowest-energy structure system, the hydrogen atom is more conducive to adsorption to the apex.

The D3 Grimme correction [50] significantly enhances the accuracy of DFT calculations by accounting for dispersion interactions, which are often neglected in traditional DFT but crucial for describing intermolecular forces in systems like van der Waals complexes. To investigate the impact of D3 Grimme correction on the calculation results, we recalculated the structures listed in Figure 1, Figure 2 and Figure 3 using D3 Grimme correction and Double-Zeta Plus Polarization (DZP) basis set, D3 Grimme correction and Triple-Zeta Plus Polarization (TZP) basis set, respectively. The calculated binding energy data are listed in parentheses in Table 1, Table 2 and Table 3. The first item in parentheses is for the DZP basis group, and the second item is for the TZP basis group. We found that although there were slight changes in the binding energy data obtained, the geometric structure remained nearly unchanged, the energy order between several stable structures of the same cluster remained unchanged, and the lowest-energy structure remained unchanged. Except for one exception, the lowest energy of Al3H2 after D3 correction is Al3H2b. But the energy difference calculated for Al3H2a and Al3H2b in these three cases is very small. This indicates that for small aluminum clusters, the conclusions drawn using TZP and DZP basis sets are almost consistent. In D3 Grimme correction, all structures have been optimized, except for Al8a in Figure 1 and Al7H2a and Al8H2a in Figure 2. These three structures are very prone to deformation in geometric optimization, so we only calculated the single point energy of these three structures.

### 2.4. Electronic Properties

Atomic averaged binding energy (E_b_), ionization potential (IP), electron affinities (EAs), chemical hardness, and the highest occupied orbital and the lowest unoccupied orbital (HOMO-LUMO) gap (all in eV) for the stable planarAl_n_ clusters are all shown in Table 1. For the stable Al_n_H_m_ (n = 3–8, m = 1, 2),the clusters are all shown in Table 2. For the lowest-energy Al_n_H_m_ (n = 6–10, m = 0–2),the clusters are all shown in Table 3.

The binding energy per atom (E_b_) is defined here as

(1) $$E_{b}(Al_{n}H_{m})=\frac{E(Al_{n}H_{m})-n\times E(Al)-m\times E(H)}{n+m}$$

**Figure 4:**
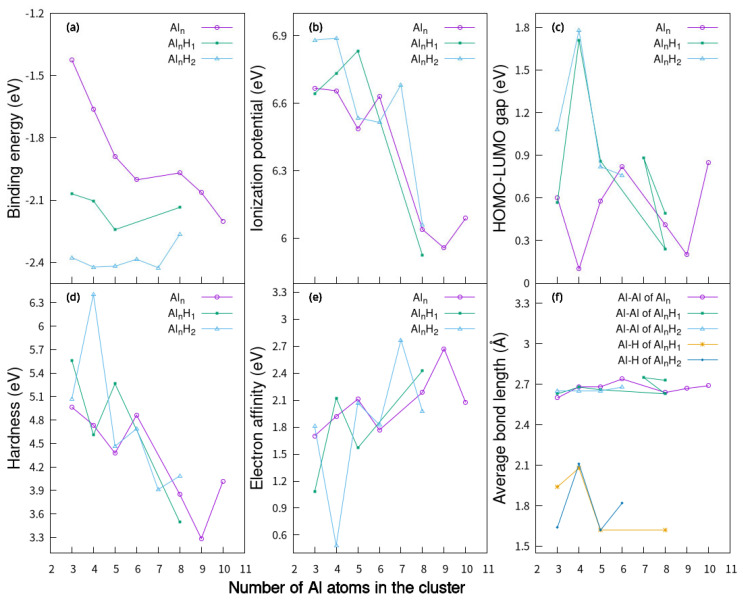
(**a**) Binding energy per atom, (**b**) ionization potential, (**c**) HOMO-LUMO gap, (**d**) chemical hardness, (**e**) electron affinity, (**f**) the average bond lengths of Al_n_H_m_ planar clusters (n = 3–10, m = 1–2).

**Figure 5:**
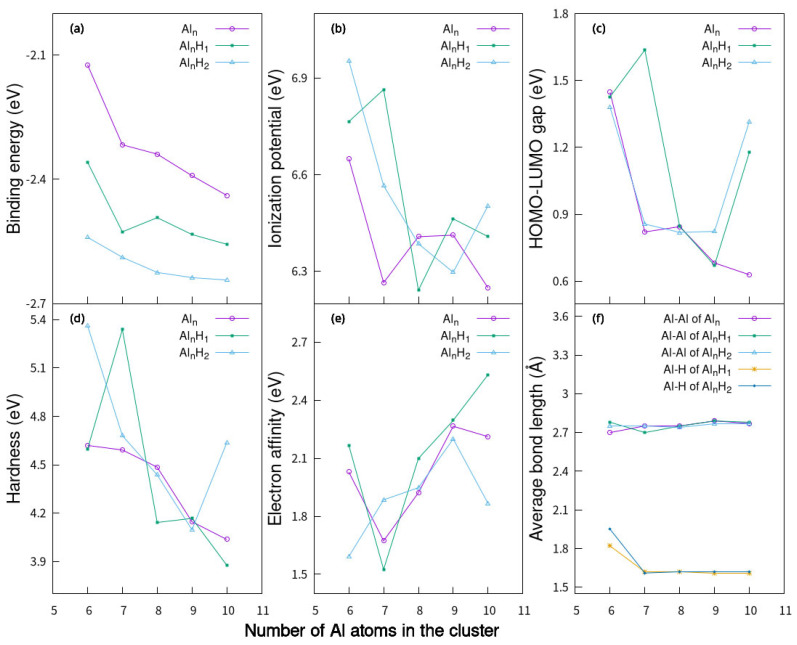
(**a**) Binding energy per atom, (**b**) ionization potential, (**c**) HOMO-LUMO gap, (**d**) chemical hardness, (**e**) electron affinity, (**f**) the average bond lengths of the lowest-energy Al_n_H_m_ clusters (n = 6–10, m = 0–2).

In Equation (1), E (Al_n_H_m_) is the total energy for Al_n_H_m_ clusters (n = 3–10, m = 0–2), E (H) or E (Al) is the single atom energy for pure clusters. The binding energy per atom $E_{b}(Al_{n}H_{m})$ of Al_n_H_m_ structures in Figure 1 and Figure 2 as a function of the number of Al atoms in the cluster is shown in Figure 4a. For small planar Al_n_ clusters (n = 3–10, n ≠ 7), the binding energy decreases gradually with the increase in n, except that at Al_8_, it is slightly larger than that at Al_7_. When a hydrogen atom is added to planar Al_n_ (n = 3–5, 8)clusters, their binding energies are in the range of −2.07 eV to −2.13 eV. When two hydrogen atoms are added to the planar Al_n_ (n = 3–8) clusters, the binding energies are in the range of −2.27 eV to −2.47 eV. The binding energy per atom $E_{b}(Al_{n}H_{m})$ of Al_n_H_m_ (n = 6–10, m = 1–2) structures in Figure 3 as a function of the number of Al atoms in the cluster is shown in Figure 5a. We can see that for Al_n_, Al_n_H_1_ and Al_n_H_2_ with the lowest-energy structure, the binding energy decreases with an increase in aluminum atoms. This is consistent with the decreasing trend of the binding energy of planar aluminum clusters.

The ionization potential and electron affinity are defined as

(2) $$IP(n)=E(Al_{n}{H_{m}}^{+})-E(Al_{n}H_{m}),$$

(3) $$EA(n)=E(Al_{n}H_{m})-E(Al_{n}H_{m}^{-}),$$

In calculating IP and EA, only the single point energy of Al_n_H_m_^+^ and Al_n_H_m_^−^ with the same geometric structure as neutral is calculated. There have been many reports on the photoelectron spectroscopy and other experiments of aluminum clusters in the literature [41,51,52,53,54]. We can first compare the EA and IP of Al3a-Al5a in Table 1 and Al6-Al10 in Table 3 with experimental data, which can verify the consistency between our calculation results and experiments. The experimental data of the IP provided in Table 1 in the literature [41] and the VIP experimental data in Table 1 in the literature [40] are in good agreement with the IP data we calculated. The average difference between experimental and theoretical IP values is 0.13 eV and 0.08 eV, respectively. The experimental data of VDE provided in Table 1 in the literature are roughly consistent with the EA data we calculated. The average difference between experimental and theoretical values is 0.44 eV. Formula 5 in the literature [40] indicates that VDE is the difference between the energy of the optimized anionic cluster and the neutral single point energy of the same geometric structure, while our theoretically calculated EA is the difference between the energy of the optimized neutral cluster and the single point energy of the anions of the same structure. The structure of the optimized anionic cluster may differ slightly from that of the optimized neutral cluster, which may be due to the average difference of 0.44 eV above.

Figure 4b,e show the variation in IP and EA with increasing cluster sizes of AlnHma (n = 3–10, m = 0–2). The overall trends of IP curves of AlnHma (m = 0–2)are downward. The general trend of the EA curve fluctuates and increases. The variation trend of IP and EA with size is the same as that of IP and EA with size in the P_n−1_Al (n = 20–40) cage [55]. Figure 5b,e show the variation in IP and EA with increasing cluster sizes of the lowest-energy Al_n_H_m_ (n = 6–10, m = 0–2), respectively. After Al_6_ and Al_10_ adsorb hydrogen atoms, their IP value will increase, and it will be higher after adsorbing two hydrogen atoms. But, for Al_7_, Al_9_ has a higher IP for adsorbing a hydrogen atom. Compounds with high electron affinity are relatively more stable. When absorbing a hydrogen atom, the electron affinity of Al4H1a with planar structure is 0.2 eV greater than that of Al4a, Al8H1a with planar structure is 0.24 eV greater than that of Al8a with planar structure, and 0.27 eV greater than that of Al8 with a stereoscopic structure. When absorbing two hydrogen atoms, the electron affinity of Al3H2a with a planar structure is 0.11 eV greater than that of Al3a, and that of Al6H2a with a planar structure is 0.06 eV greater than that of Al6a with a planar structure. This indicates that at least the structures of these planar aluminum clusters adsorbed with hydrogen atoms can exist stably. The change in cluster size is only one factor that affects properties such as IP and EA. These properties will vary with each atom in a nonintuitive way, and the properties of clusters are influenced by many factors.

The hardness (η) is defined as

(4) η=IP−EA.

The large value of η indicates the greater stability of the cluster. From Figure 4d, the general trend of the hardness curve of AlnHma (m = 0–2) also decreases. This is consistent with the decreasing trend of the hardness of the lowest-energy aluminum clusters. Compared with Figure 4d and Figure 5d, we found that the hardness of the planar structure and the hardness of the lowest-energy structure have the same downward trend. The maximum hardness value appears in Al4H2 (6.41 eV), while the minimum hardness value appears in the planar Al_9_ cluster (3.29 eV).

The highest occupied orbital and the lowest unoccupied orbital (HOMO-LUMO) energy gap curves are shown in Figure 4c and Figure 5c as a function of the cluster size. The HOMO-LUMO gap is used to elucidate the relative stabilities of clusters. The larger HOMO-LUMO energy gap indicates electronic stability; that is, the cluster is neither willing to give nor accept charges. Clusters with larger HOMO-LUMO gaps are more stable and with higher chemical inertness. For planar Al_n_ (n = 3–10), the gap value of Al_4_ is the smallest, while for Al_n_H_1_ and Al_n_H_2_ in this study, the gap value of Al_4_H_1_ and Al_4_H_2_is the highest. Al_4_H_1_ and Al_4_H_2_ should have large abundances. The large HOMO-LUMO gap of Al_4_H_1_ and Al_4_H_2_ is likely to make the further interaction of hydrogen with these clusters energetically not so favorable. In our calculations, the HUMO-LUMO gaps of other Al_n_H_m_ clusters are smaller than those of Al_4_H_1_ and Al_4_H_2_, which may mean that their existence is not as abundant as Al_4_H_1_ and Al_4_H_2_. It has been reported in the literature [25] that Al_4_H_7_^−^ has a very stable existence; Al_n_H_m_ has magic number clusters [14]. Although Al_4_H_1_ and Al_4_H_2_ do not meet the conditions for magic numbers, their HOMO-LUMP gap is relatively high, indicating that the clusters in the Al_4_H_m_ series are relatively stable. The reason why the HOMO-LUMO gap of Al_4_ after absorbing one or two hydrogen atoms is larger than that of adjacent Al_3_ and Al_5_ after absorbing the same hydrogen atoms can be explored from the following aspects. Firstly, the geometric structure of aluminum clusters is different. Al4a in Figure 1 is rectangular, while Al3a is triangular, and Al5a can be regarded as composed of two triangles. Different geometric structures have varying impacts on the HOMO-LUMO gap. Secondly, the HOMO-LUMO gap is related to the adsorption position of hydrogen atoms, because different adsorption sites imply different electron arrangements and interactions between aluminum and hydrogen. In the structures shown in Figure 2, hydrogen is located at the four-fold position in Al4H1a, the top position in Al4H1b, and the bridge position in Al4H1c. Their HOMO-LUMO gaps are 1.71 eV, 0.86 eV, and 0.63 eV, respectively. Two hydrogen atoms are located at opposite four-fold positions in Al4H2a, at bridge and top positions in Al4H2b and Al4H2c, at four-fold and bridge positions in Al4H2d, and at bridge and top positions in Al4H2e. Their HOMO-LUMO gaps are 1.78 eV, 0.79 eV, 0.72 eV, 1.45 eV, and 0.60 eV, respectively. From these data, we can see that the HOMO-LUMO gap of hydrogen atoms located at the four-fold position is higher than that located at the bridge and top positions. Similar conclusions have been drawn for Al3, where two hydrogen atoms in Al3H2b are located at opposite three-fold positions with a HOMO-LUMO gap of 1.75 eV, while two hydrogen atoms in Al3H2a are located at two top positions with a HOMO-LUMO gap of 1.08 eV. The HOMO-LUMO gaps for Al5H2a to Al5H2e in Figure 2 are 0.82 eV, 0.44 eV, 0.33 eV, 0.98 eV, and 0.31 eV, respectively. Their hydrogen atoms are mainly located at the top or bridge positions. The above data indicate that in the competition for different adsorption positions, the HOMO-LUMO gap value corresponding to the four-fold position is the highest.

From Figure 4e and Figure 5e and Table 1, Table 2 and Table 3, the average bond lengths of Al-Al and Al -H in Al_n_H_m_ can be obtained. Whether it is a planar cluster of pure aluminum or Al_n_H_1_ and Al_n_H_2_ adsorbed hydrogen atoms, the bond length of Al-Al ranges from 2.60 Å to 2.74 Å, indicating that hydrogen adsorption has a limited influence on the bond length of Al-Al. In our calculations, the average bond length of Al-Al in Al_n_H_m_ (n = 6–10, m = 0–2) with the lowest-energy structure ranges from 2.70 Å to 2.79 Å. The maximum change in the average bond length after the adsorption of the hydrogen atom is 0.08 Å. This also confirms the conclusion of the previous plane structure, in that the effect of hydrogen adsorption on the bond length of Al is limited.

From Figure 2, we can see that the length of the Al-H bond is closely related to the adsorption position of the H atom. From Figure 2, we can see that when the hydrogen atom is at the top site, the bond length of Al-H ranges from 1.62 Å to 1.64 Å, with almost no change in length; when the hydrogen atom is at the bridge site, the Al-H bond length ranges from 1.73 Å to 1.96 Å, with an average value of 1.82 Å; when the hydrogen atom is located in the three-fold site, the Al-H bond length ranges from 1.88 Å to 2.06 Å, with an average value of 1.95 Å; when the hydrogen atom is located at the four-fold site, the Al-H bond length ranges from 2.08 Å to 2.11 Å. The Al-H bond located at the top site has the smallest length, followed by the bridge site, followed by the three-fold site, and the long bond is located at the four-fold site. When the adsorption position of hydrogen is the same, the bond length of Al-H remains almost unchanged in clusters of different sizes, indicating that the Al-H bond has a similar nature in different clusters. The general trend of the Al-H bond length is consistent with the report by Kawamura et al. [49]. These trends can also be confirmed in Al_n_H_m_ (n = 6–10, m = 0–2) with the lowest-energy structure. The hydrogen atom in Al_6_H_1_ with the lowest energy is adsorbed at the bridge site, and its bond length is 1.82 Å. Two hydrogen atoms in Al_6_H_2_ are adsorbed at the three-fold site, and their average bond length is 1.95 Å. In addition, in other structures, the hydrogen atom is adsorbed at the top position, and its bond length is 1.61 Å or 1.62 Å.

**Figure 6:**
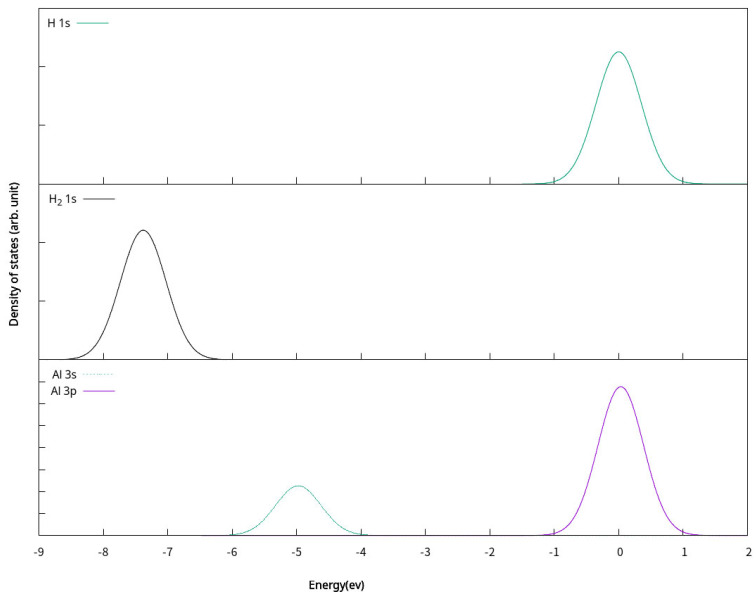
Partial density of states(PDOS) of H atom, H_2_ molecule and Al atom. The Fermi energy is shifted to zero.

**Figure 7:**
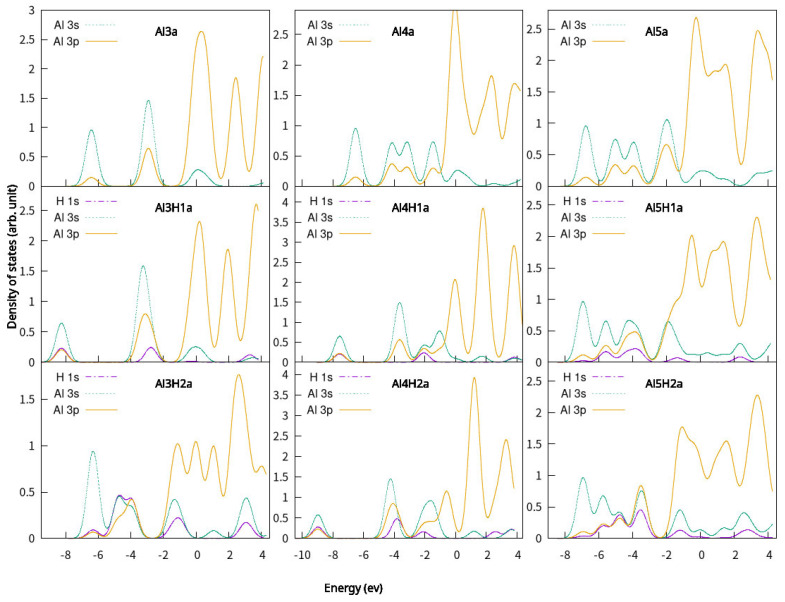
Partial density of states (PDOS) of Al_n_H_m_ (n = 3–5, m = 1–2). The Fermi energy is shifted to zero.

To further understand the bonding of the clusters and its H-bonded complexes, the partial density of states (PDOS) of the H atom, Al atom, the isolated H_2_ molecule, clusters, and their one H or two H bonded complexes was calculated. The PDOS plots are shown in Figure 6 and Figure 7 with the Femi energy levels fixed at 0 eV, respectively. By comparing the PDOS of Al_n_ (n = 3–5) planar clusters and their corresponding one H- or two H-bonded complex, an orbital overlap between Al and H atoms is observed. The s electrons of H in Al_n_H_m_ (n = 3–5, m = 1–2) are distributed similar to those of Al s and p electrons. Thus, the s-s and s-p hybridization is the dominant interaction between H and Al. It indicates the interaction between the clusters and one H atom or two H atoms. This results in the chemical interaction between an H atom and Al_n_ (n = 3–5) planar clusters.

**Figure 8:**
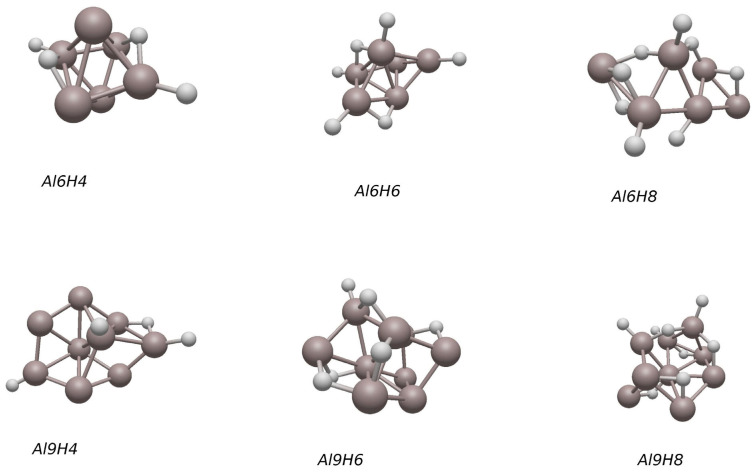
The lowest-energy structures of Al_n_H_2m_ clusters (n = 6,9, m = 2–4). The white small ball represents hydrogen atoms.

We also calculated the adsorption of multiple hydrogen atoms on aluminum clusters. Figure 8 shows the adsorption of four, six, and eight hydrogen atoms on Al_6_ and Al_9_. Figure 8 was drawn using Avogadro^2^ (version 1.93.0) software [56]. When Al_6_ adsorbs four hydrogen atoms, the structure of its aluminum clusters changes very little compared to the structure of aluminum in Al_6_H_1_ and Al_6_H_2_; when eight hydrogen atoms are adsorbed, the structure of the aluminum cluster undergoes significant deformation. In Al_6_ adsorption, the hydrogen atoms are mainly located at the top and bridge positions. The same situation also occurs when Al_9_ clusters adsorb hydrogen atoms. After adsorbing four, six, and eight hydrogen atoms, the structure of Al_9_ also underwent significant deformation, and these hydrogen atoms were mainly located at the top and bridge positions.

In order to study the adsorption process of H_2_ on Al clusters, we calculated the adsorption energy of H_2_ in the dissociative adsorption configuration. The adsorption energy for dissociative adsorption configurations is calculated as follows [1]

(5) $$E_{\mathrm{Ad}}=\left(Al_{n}-m\times H\right)=E(Al_{n})+E(H_{2})-E(Al_{n}H_{2m})$$

The results are listed in Table 4. Adsorption energy represents the minimum energy required for the separation of an adsorbate from the cluster surface. The structure of Al_n_ in the formula depends on whether the substructure Al_n_ in Al_n_H_2_ is planar or three-dimensional. In our calculation, the adsorption energy of planar aluminum clusters is between 0.44 eV and 1.43 eV. Even though the adsorption energy of Al_4_ is the largest in planar clusters, the overall trend is that the adsorption energy decreases with an increase in cluster size. When adsorbing two hydrogen atoms, the absorption energy of the three-dimensional aluminum cluster is between 0.62 eV and 1.12 eV. Even though the adsorption energy of Al_7_ clusters is the smallest of all three-dimensional clusters, the overall trend is that the adsorption energy decreases with an increase in cluster size when adsorbing two hydrogen atoms. The decrease in the adsorption barrier may be due to the increase in average binding energy with the increase in cluster size [1]. When the number of aluminum clusters remains constant and the number of hydrogen atoms adsorbed by Al_6_ increase, the general trend of the adsorption energy is to increase, reaching a peak of 2.75 eV at Al_6_H_6_. When Al_9_ adsorbs multiple hydrogen atoms, the adsorption energy gradually increases.

**Figure 9:**
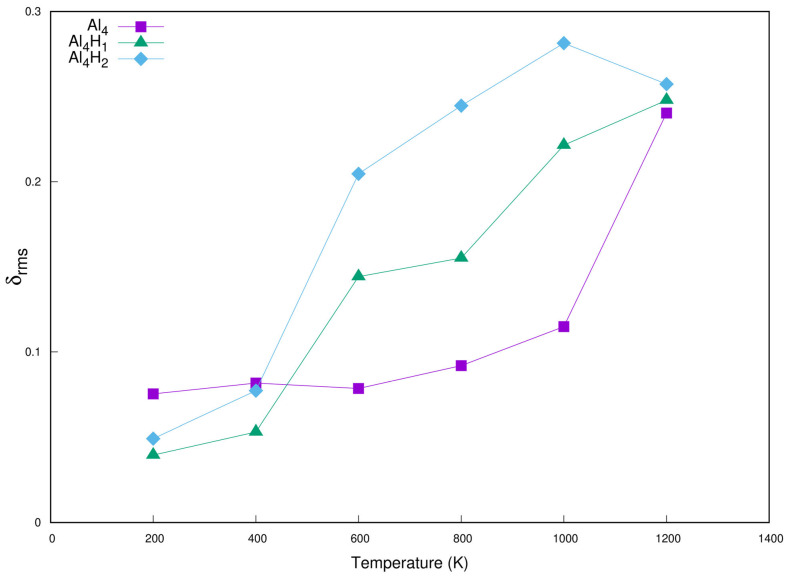
δ_rms_ as a function of temperature.

In order to further study the stability of the cluster, we performed DFT-based molecular dynamics calculations of the Al_4_, Al_4_H_1_, Al_4_H_2_clusters at finite temperature. The simulations were carried out with the Al4a, Al4H1a, Al4H2a structures listed in Figure 1 and Figure 2as the starting conformations at temperatures of 200 K, 400 K, 600 K, 800 K, 1000 K and 1200 K. We used the ground state structures Al4a, Al4H1a, and Al4H2a in Figure 1 and Figure 2 as the starting configurations for molecular dynamics calculations. For Al_4_, at 800 K, the final structure evolved into distorted Al4a, and at 1000 K, it finally evolved into a three-dimensional configuration. For the Al_4_H_1_ cluster, at 600 K, the final structure evolved into Al4H1c, as shown in Figure 2, and at 1200 K, it finally evolved into Al4H1b. At 600 K, the Al_4_H_2_ cluster evolved into the final structure of Al4H1b, and at higher temperatures, some distorted structures appeared. As De et al. [57] pointed out, clusters may have a solid-like state, a liquid-like state, or a state of transformation from solid-like to liquid-like at different temperatures. The solid-like state has more structural stability than the liquid-like state. The root mean square bond length fluctuations (δ_rms_) are plotted as a function of temperature in Figure 9. During the transition of clusters from a solid to liquid state, the δ_rms_ value will significantly increase. We can see from Figure 9 that for Al_4_H_1_ and Al_4_H_2_, the δ_rms_ value increases sharply in the temperature range of 400 K to 600 K, and for Al4, the δ_rms_ value increases sharply in the temperature range of 1000 K to 1200 K. At temperatures below 400 K, the delta values of these clusters are very small, and the atoms mainly vibrate near the equilibrium position. At this point, the clusters are in a solid state, and the overall structure of the clusters remains stable.

We can see the energy difference between each structure and the lowest-energy structure from Figure 1 and Figure 2. The planar structure of Al6a has an energy 0.73 eV higher than the ground-state structure, while Al8a has an energy 2.96 eV higher, Al9a has an energy 2.96 eV higher, Al10a has an energy 2.38 eV higher, Al8H1a has an energy 3.22 eV higher, Al6H2a has an energy 1.25 eV higher, Al7H2a has an energy 1.47 EV higher, and Al8H2a has an energy 3.60 eV higher. As the atomic size increases, the number of metastable configurations of atomic clusters also increases, among which planar clusters also exist. These planar clusters were only discovered after we took large-scale initial samples. Although they are thermodynamically disadvantageous compared to the ground state, they may exist in excited or transition states. In our molecular dynamics calculations of Al_4_, Al_4_H_1_, and Al_4_H_2_, we discovered metastable structures of Al_4_, Al_4_H_1_, and Al_4_H_2_, at higher temperatures.

The stability of clusters can also be studied from the possibility of cluster transformation reactions. Al_8_ may dissociate into two Al_4_, and Al_8_H_2_ may dissociate into two Al_4_H_1_. The behavior of dissociation is related to dissociation energy [40]. For the dissociation channels mentioned above, we can define dissociation energy as follows:

(6) $$DE=E(Al_{n}H_{m})-E(Al_{n-l}H_{m-k})-E(Al_{l}H_{k}),$$

We will use the dissociation of Al_8_ as an example to discuss. If Al8a in Figure 1 dissociates into Al3a and Al5a in Figure 1, then the dissociation energy is 2.03 eV. If Al8a in Figure 1 decomposes into two Al4a in Figure 1, then the dissociation energy is 2.46 eV. If Al8 in Figure 3 dissociates into two Al4a in Figure 1, then the dissociation energy is 5.43 eV. If Al8H2 in Figure 3 is dissociated into two Al4H1a in Figure 1, then its dissociation energy is 5.21 eV. In the above cases, the dissociation energy is positive, indicating that the dissociation process is thermodynamically unfavorable. The absolute value of dissociation energy in the third case is larger than that in the first and second cases, indicating that Al8 in Figure 3 is more stable than Al8a in Figure 1. The energy difference between Al8H4 and two Al4H2a in Figure 2 is −3.90 eV. This indicates that the process of synthesizing two Al_4_H_2_ into one Al_8_H_4_ requires the absorption of heat, which is thermodynamically disadvantageous. If a suitable temperature and pressure environment can be created, catalysts may be used to synthesize Al_8_H_4_ from Al_4_H_2_ in the future.

**Figure 10:**
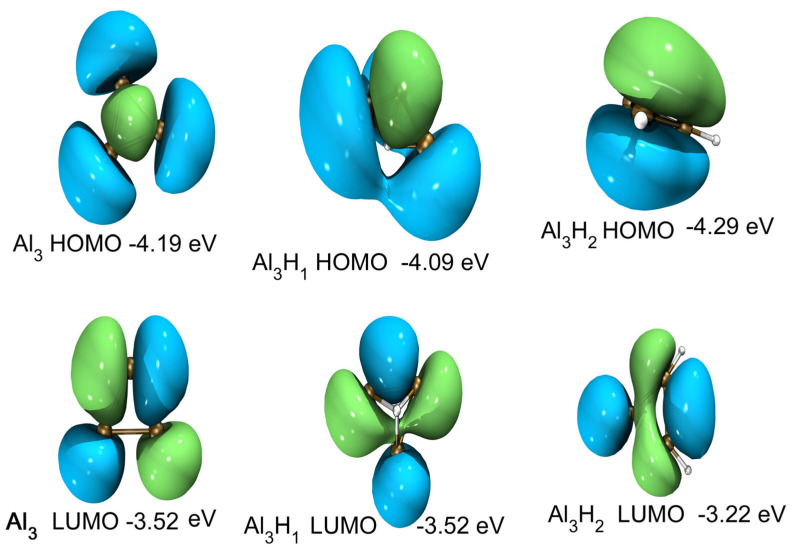
HOMO and LUMO diagramand orbital energy levels of Al_3_,Al_3_H_1_ and Al_3_H_2_. The isosurface value is 0.05 e/Å^3^.

**Figure 11:**
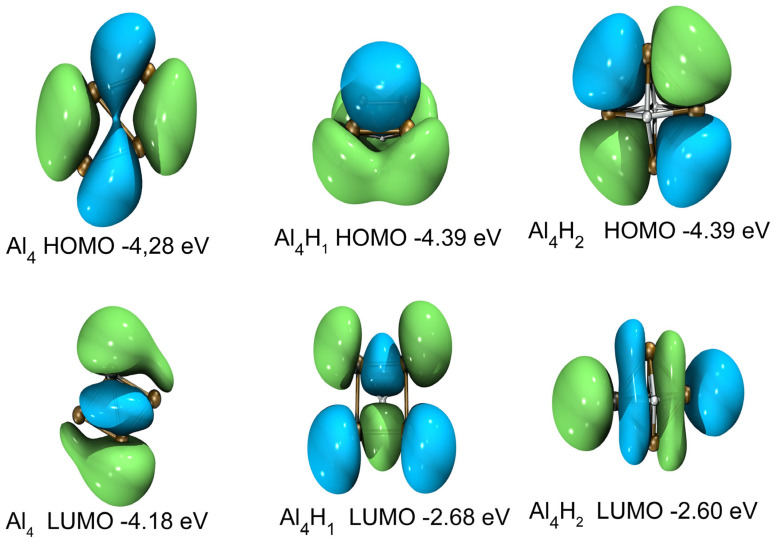
HOMO and LUMO diagram and orbital energy levels of Al_4_,Al_4_H_1_ and Al_4_H_2_. The isosurface value is 0.05 e/Å^3^.

**Figure 12:**
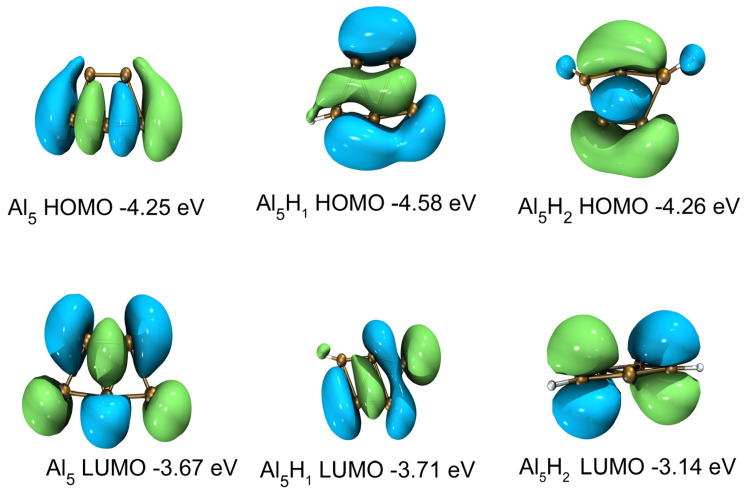
HOMO and LUMO diagram and orbital energy levels of Al_5_,Al_5_H_1_ and Al_5_H_2_. The isosurface value is 0.05 e/Å^3^.

Figure 10, Figure 11 and Figure 12 show the distributions of HOMO and LUMO of Al_n_H_m_ (n = 3–5, m = 0–2) clusters, respectively. HOMO and LUMO diagrams are drawn using VMD (version 1.9.3)software [58]. The technology introduced by Lu et al. [59] is used in the drawing of HOMO and LUMO. We can see almost all aluminum atoms contribute to HOMO and LUMO in Al_n_H_m_ (n = 3–5, m = 0–2) clusters. The delocalization of the frontier orbital is obvious in Al_n_ (n = 3–5) planar clusters. After binding with one or two hydrogen atoms, the shape of HOMO and LUMO of Al_n_H_m_ (n = 3–5, m = 0–2) clusters is changed, which indicates that adding a hydrogen atom has a strong influence on the frontier orbital. The contribution of hydrogen appears in the HOMO and LUMO of Al_n_H_m_ (n = 3–5, m = 0–2) clusters.

The aromaticity of clusters has been extensively studied [60,61,62,63,64]. Aluminum and boron are elements of the same main group, and boron-related clusters are mostly typical three-dimensional aromatic species [65]. The Al_4_^2−^ dianion is a well-known two-dimensional aromatic species [66]. The aluminum clusters in this study have both two-dimensional planar and three-dimensional structures. Therefore, studying the aromaticity of these aluminum clusters and the conversion between two-dimensional and three-dimensional aromaticity is a very interesting question that deserves detailed research in the future.

## 3. Materials and Methods

In this work, the initial structures of planar aluminum clusters Al_n_ (n = 3–10)are generated randomly. All initial planar cluster structures were fully relaxed with SIESTA (version 3.1,in addition, version 5.2.1 was used for D3 Grimme correction) software package [67] without any symmetry constraint. An optimized double-ζ basis set with polarization orbital [DZP] [68], GGA with the Revised Perdew–Burke–Ernzerhof (RPBE) functional [69] and norm-conserving Troullier–Martins pseudopotentials [70] were adopted. In our calculations, all the Al_n_ clusters (n = 3–10) were placed in a cubic cell 15 Å (for n = 3) or 20 Å (for n = 4–7) or more than 30 Å (for n ≥ 8). We used a mesh cutoff energy of 180 Ry to determine the self-consistent charge density, which provided us with a precision in total energy of ≤2 meV/atom. All geometries were optimized by SIESTA using the conjugate gradient method [71], until none of the residual Hellmann–Feynman forces exceeded 10^−3^ eV/Å.

After obtaining the structure of stable planar aluminum clusters using SIESTA, hydrogen adsorption sites are randomly generated on these stable planar aluminum clusters. The initial structure of hydrogen adsorption on planar aluminum clusters was obtained. The corresponding equilibrium Al–Al bond length of 2.85Å for Al_2_ from our calculations using GGA-RPBE functional coincides well with experiment data 2.835 Å [72]. The calculated H–H bond length is 0.79 Å for H_2_. The calculated Al–H bond length is 1.71 Å for AlH. Our previous work on Al_n_P_13−n_ (n = 0–13) clusters [73] provided a detailed introduction to the influence of different functional choices on the calculation results when calculating aluminum-related clusters. The use of RPBE functional and DZP basis function can achieve good calculation results.

To study the stability of clusters, the lowest-energy conformation is chosen as the starting conformation for the molecular dynamical (MD) simulations. The finite temperature simulation for the lowest-energy cluster is carried out implementing SIESTA code using the same exchange–correlation functional (GGA/RPBE) as described above. The simulations are carried out between 200 and 1200 K. The nuclear positions are updated using Nose MD with a time step of 1fs. The temperature in Nose MD is controlled by means of a Nosé thermostat. The atomic positions and bond length fluctuations of atoms are analyzed using root mean square bond length fluctuations (δ_rms_). The δ_rms_ is defined as [57]

(7) $$\delta_{rms}=\frac{2}{N(N-1)}\sum_{i<j}\frac{\sqrt{{\langle {R_{ij}}^{2}\rangle }_{t}-{{\langle R_{ij}\rangle }^{2}}_{t}}}{{\langle R_{ij}\rangle }_{t}}$$

where N is the number of particles in the system, R_ij_ is the distance between the ith and jth particle in the system and ${\langle \ldots \rangle }_{t}$ denotes a time average over the entire trajectory.

## 4. Conclusions

In this work, the low-lying energy structures of small planar aluminum clusters Al_n_ (n = 3–6, 8–10) and hydrogenated small planar aluminum clusters Al_n_H_m_ (n = 3–8, m = 1–2) are determined by DFT calculations. Many stable planar structures have been found; some are consistent with the reported ones, and some are new configurations. Low-lying-energy small planar aluminum clusters are mostly composed of triangles. Compared with the planar pure aluminum cluster structure, the structure of aluminum atoms in the hydrogenated cluster remains almost unchanged. Hydrogen is adsorbed at different positions on planar aluminum clusters. When the number of aluminum atoms n is greater than six, the lowest-energy structure of Al_n_H_m_ (n = 6–10, m = 0–2) is also calculated and compared with the planar structure. Dissociative adsorption configurations of the planar structure and lowest-energy structure experienced a decrease in hydrogen adsorption energy with an increase in cluster size. The E_b_, IP, EA, hardness, HOMO-LUMO gap, HOMO, LUMO orbital, PDOS and average bond length were discussed. Among the clusters we calculated, Al_4_H_1_ and Al_4_H_2_ have the highest HOMO-LUMO gap, indicating that they may be more abundant than other clusters. The stability of clusters was discussed using DFT-based finite temperature molecular dynamics.

## Figures and Tables

**Table 1:** The average bond lengths $d_{\mathrm{Al}-\mathrm{Al}}$, binding energies (BEs), the BE values in parentheses use the D3 Grimme method, DZP basis function, the D3 Grimme method, and TZP basis function, respectively, as well as ionization energy (IP), electron affinity (EA), chemical hardness η and HUMO-LUMO gap of the low-energy structures of planar aluminum clusters. $d_{\mathrm{Al}-\mathrm{Al}}$ is the mean nearest-neighbor bond lengths between aluminum atoms.

Cluster	$d_{Al-Al}$ (Å)	BE (eV)	IP (eV)	EA (eV)	η (eV)	Gap (eV)
Al3a	2.60	−1.42 (−1.47, −1.50)	6.67	1.70	4.96	0.60
Al4a	2.68	−1.66 (−1.72, −1.74)	6.66	1.92	4.74	0.10
Al5a	2.68	−1.89 (−1.95, −1.97)	6.49	2.11	4.38	0.58
Al6a	2.74	−2.00 (−2.07, −2.08)	6.63	1.77	4.86	0.82
Al8a	2.64	−1.97 (−2.03, −2.05)	6.04	2.19	3.85	0.41
Al9a	2.67	−2.06 (−2.13, −2.15)	5.96	2.67	3.29	0.20
Al10a	2.69	−2.20 (−2.27, −2.29)	6.09	2.07	4.02	0.85

**Table 2:** The average bond, the average bond lengths $d_{\mathrm{Al}-\mathrm{Al}}$, $d_{\mathrm{Al}-H}$, binding energies (BEs). The BE values in parentheses use the D3 Grimme method, DZP basis function, the D3 Grimme method, TZP basis function, respectively, and the ionization energy (IP), electron affinity (EA), chemical hardness (η) and HUMO-LUMO gap of the low-energy structures of hydrogenated aluminum clusters. $d_{\mathrm{Al}-\mathrm{Al}}$ and $d_{\mathrm{Al}-H}$ are the mean nearest-neighbor bond lengths between aluminum atoms and hydrogen and aluminum atoms. Location of H is represented by symbols 3, 4, n, o, b and t, which mean three- and four-fold, neighboring, opposite, bridge, and top site, respectively.

Cluster	$d_{Al-Al}$ (Å)	$d_{Al-H}$ (Å)	BE (eV)	IP (eV)	EA (eV)	η (eV)	Gap (eV)	Location of H
Al3H1a	2.63	1.94	−2.07 (−2.11, −2.14)	6.64	1.08	5.56	0.57	3
Al4H1a	2.68	2.08	−2.10 (−2.16, −2.18)	6.73	2.12	4.61	1.71	4
Al5H1a	2.66	1.62	−2.24 (−2.30, −2.31)	6.83	1.57	5.26	0.86	t
Al8H1a	2.63	1.62	−2.13 (−2.19, −2.21)	5.92	2.43	3.49	0.24	t
Al3H2a	2.65	1.64	−2.38 (−2.41, −2.43)	6.88	1.81	5.07	1.08	t, t
Al4H2a	2.65	2.11	−2.42 (−2.48, −2.51)	6.89	0.48	6.41	1.78	4, o
Al5H2a	2.65	1.62	−2.42 (−2.47, −2.48)	6.53	2.07	4.47	0.82	t, t
Al6H2a	2.68	1.82	−2.38 (−2.44, −2.46)	6.52	1.83	4.69	0.76	t, n
Al7H2a	2.75	1.62	−2.43 (−2.49, −2.50)	6.68	2.77	3.91	0.88	t, n
Al8H2a	2.73	1.94	−2.27 (−2.32, −2.44)	6.06	1.97	4.09	0.49	3, o

**Table 3:** The average bond lengths $d_{\mathrm{Al}-\mathrm{Al}}$, $d_{\mathrm{Al}-H}$, binding energies (BEs). The BE values in parentheses use the D3 Grimme method, DZP basis function, the D3 Grimme method, TZP basis function, respectively, and the ionization energy (IP), electron affinity (EA), chemical hardness η and HUMO-LUMO gap of the low-energy structures of hydrogenated aluminum clusters. $d_{\mathrm{Al}-\mathrm{Al}}$ and $d_{\mathrm{Al}-H}$ are the mean nearest-neighbor bond lengths between aluminum atoms and hydrogen and aluminum atoms.

Cluster	$d_{Al-Al}$ (Å)	$d_{Al-H}$ (Å)	BE (eV)	IP (eV)	EA (eV)	η (eV)	Gap (eV)	Location of H
Al6	2.70		−2.12 (−2.19, −2.21)	6.55	2.03	4.62	1.45	
Al6H1	2.78	1.82	−2.36 (−2.43, −2.45)	6.76	2.17	4.60	1.43	B
Al6H2	2.75	1.95	−2.54 (−2.61, −2.64)	6.95	1.59	5.36	1.38	3, 3
Al7	2.75		−2.32 (−2.39, −2.43)	6.26	1.67	4.59	0.82	
Al7H1	2.70	1.62	−2.52 (−2.60, −2.63)	6.86	1.53	5.34	1.64	T
Al7H2	2.75	1.61	−2.59 (−2.66, −2.69)	6.57	1.89	4.68	0.86	t, t
Al8	2.75		−2.34 (−2.42, −2.45)	6.41	1.92	4.49	0.84	
Al8H1	2.75	1.62	−2.49 (−2.57, −2.60)	6.24	2.10	4.14	0.85	T
Al8H2	2.74	1.62	−2.63 (−2.70, −2.73)	6.39	1.95	4.44	0.82	t, t
Al9	2.79		−2.39 (−2.48, −2.51)	6.41	2.27	4.15	0.68	
Al9H1	2.79	1.61	−2.53 (−2.62, −2.65)	6.46	2.29	4.17	0.67	T
Al9H2	2.77	1.62	−2.64 (−2.72, −2.75)	6.30	2.20	4.10	0.82	t, t
Al10	2.77		−2.44 (−2.53, −2.56)	6.25	2.21	4.04	0.63	
Al10H1	2.78	1.61	−2.56 (−2.64, −2.68)	6.41	2.53	3.88	1.18	T
Al10H2	2.77	1.62	−2.64 (−2.73, −2.76)	6.50	1.87	4.64	1.32	t, t

**Table 4:** Calculated adsorption energies [Ead (eV)] for dissociated adsorption of hydrogen on Al_n_ (n = 3–10) clusters.

Structure	Ead (eV)	Structure	Ead (eV)	Structure	Ead (eV)	Structure	Ead (eV)
Al3H2a	1.15	Al_6_H_4_	1.95	Al_8_H_2_	1.08	Al9H8	1.28
Al4H2a	1.43	Al_6_H_6_	2.75	Al_8_H_4_	1.34	Al10H2	0.87
Al5H2a	1.02	Al_6_H_8_	2.14	Al_9_H_2_	1.03		
Al6H2a	0.61	Al_7_H_2_	0.62	Al_9_H_4_	1.15		
Al6H2	1.12	Al_8_H_2_a	0.44	Al_9_H_6_	1.28		

## Data Availability

Data are contained within the article.
